# Dantrolene-Induced Inhibition of Skeletal L-Type Ca^2+^ Current Requires RyR1 Expression

**DOI:** 10.1155/2013/390493

**Published:** 2012-12-05

**Authors:** R. A. Bannister

**Affiliations:** Division of Cardiology, Department of Medicine, University of Colorado Denver, Anschutz Medical Campus, P-15 8006, B-139, Aurora, CO 80045, USA

## Abstract

Malignant hyperthermia (MH) is a pharmacogenetic disorder most often linked to mutations in the type 1 ryanodine receptor (RyR1) or the skeletal L-type Ca^2+^ channel (Ca_V_1.1). The only effective treatment for an MH crisis is administration of the hydantoin derivative Dantrolene. In addition to reducing voltage induced Ca^2+^ release from the sarcoplasmic reticulum, Dantrolene was recently found to inhibit L-type currents in developing myotubes by shifting the voltage-dependence of Ca_V_1.1 channel activation to more depolarizing potentials. Thus, the purpose of this study was to obtain information regarding the mechanism of Dantrolene-induced inhibition of Ca_V_1.1. A mechanism involving a general depression of plasma membrane excitability was excluded because the biophysical properties of skeletal muscle Na^+^ current in normal mouse myotubes were largely unaffected by exposure to Dantrolene. However, a role for RyR1 was evident as Dantrolene failed to alter the amplitude, voltage dependence and inactivation kinetics of L-type currents recorded from *dyspedic* (RyR1 null) myotubes. Taken together, these results suggest that the mechanism of Dantrolene-induced inhibition of the skeletal muscle L-type Ca^2+^ current is related to altered communication between Ca_V_1.1 and RyR1.

## 1. Introduction 

In skeletal muscle, depolarization of the transverse tubule network causes conformational rearrangements within the sarcolemmal L-type Ca^2+^ channel (Ca_V_1.1) that produce a signal which is transmitted to the type 1 ryanodine receptor (RyR1) in the sarcoplasmic reticulum (SR) membrane via a transient protein-protein interaction [1]. This “orthograde” signal gates RyR1, enabling the Ca^2+^ efflux from the SR into the myoplasm which ultimately initiates contraction. In addition, RyR1 produces a “retrograde” signal that enhances Ca_V_1.1 P_o_ [2, 3] and accelerates Ca_V_1.1 activation kinetics [3–5]. Like orthograde coupling, retrograde coupling is thought to be propagated via protein-protein contracts between RyR1 and Ca_V_1.1 [5–7].

Malignant hyperthermia (MH) is a fulminant pharmacogenetic disorder in which the vast majority of identified causative mutations are found in the genes encoding RyR1 [8, 9] or Ca_V_1.1 [10–13]. MH crises are triggered by heat, depolarizing muscle relaxants, or halogenated anaesthetics [14]. Following exposure to one of these triggers, MH-susceptible individuals enter a potentially lethal hypermetabolic crisis. The only effective treatment for an MH crisis is administration of the hydantoin derivative Dantrolene, which has substantially reduced MH-related mortality since its clinical introduction in the late 1970s [15]. Despite the therapeutic success of Dantrolene, the mechanism(s) by which it ameliorates MH crises is (are) not clear. There is general agreement that one effect of Dantrolene is to stem aberrant Ca^2+^ efflux from the SR into the myoplasm that occurs during MH crises [15]. Dantrolene and its more soluble analogue azumolene have also been shown to reduce store-operated [16, 17] and voltage-triggered Ca^2+^ entry [18, 19] into muscle from the extracellular space. The major route of voltage-triggered Ca^2+^ entry into myotubes is the L-type Ca^2+^ current conducted by Ca_V_1.1 [19, 20]. In myotubes, Dantrolene reduces such Ca^2+^ entry by shifting the voltage dependence of Ca_V_1.1 activation to more depolarizing potentials [19]. Despite the aforementioned effects of Dantrolene on L-type current in mammalian muscle, the precise mechanism by which Dantrolene alters Ca_V_1.1 channel activity has not been investigated. In this study, I have sought to determine whether the previously described depolarizing shift was a consequence of a Dantrolene-induced depression in membrane excitability or a modification of bidirectional communication between RyR1 and Ca_V_1.1. In order to investigate the former possibility, the skeletal muscle Na^+^ current was employed as an assay to gauge membrane excitability. A general depression of membrane excitability appeared an unlikely explanation as Dantrolene had little effect on the biophysical properties of the Na^+^ current. To probe the latter mechanism, L-type Ca^2+^ currents were recorded from *dyspedic *(RyR1 null) myotubes. In these experiments, no Dantrolene-induced effects on the L-type current were observed, indicating a requirement for RyR1 expression in Dantrolene-induced inhibition of Ca_V_1.1.

## 2. Experimental Procedures

### 2.1. Myotube Culture

The use of mice in this study was reviewed and approved by the University of Colorado Denver-Anschutz Medical Campus Institutional Animal Care and Use Committee. Primary cultures of normal (+/+ or +/*mdg*) or *dyspedic* (RyR1 −/−) myotubes were prepared from newborn mice as described previously [21]. Myoblasts were plated onto 35 mm ECL (#08-110, Millipore, Billerica, MA)-coated, plastic culture dishes (#353801, Falcon, San Jose, CA). Cultures were grown for 6-7 days in a humidified 37°C incubator with 5% CO_2_ in Dulbecco's Modified Eagle Medium (DMEM; #15-017-CM, Mediatech, Herndon, VA), supplemented with 10% fetal bovine serum/10% horse serum (Hyclone Laboratories, Logan, UT). This medium was then replaced with differentiation medium (DMEM supplemented with 2% horse serum). Myotubes were used in experiments 3–5 days following the switch to differentiation medium.

### 2.2. Patch-Clamp Recording of Skeletal Muscle Na^+^ and L-Type Ca^2+^ Currents

Pipettes were fabricated from borosilicate glass and had resistances of ~2.0 MΩ when filled with a standard internal solution containing (mM): 140 Cs-aspartate, 10 Cs_2_-EGTA, 5 MgCl_2_, and 10 HEPES, pH 7.4 with CsOH. In order to record skeletal Na^+^ currents, the bath solution contained (mM): 140 tetraethylammonium (TEA)-Cl, 5 NaCl, 10 CaCl_2_, and 10 HEPES, pH 7.4 with TEA-OH. When recording L-type Ca^2+^ currents, the bath solution contained (mM): 145 TEA-Cl, 10 CaCl_2_, and 10 HEPES, 0.002 tetrodotoxin; pH 7.4 with TEA-OH. −P/4 and P/4 subtraction were employed to correct for linear current components while recording Na^+^ and L-type Ca^2+^ currents, respectively. Electronic compensation was used to reduce the effective series resistance and the time constant for charging the linear cell capacitance. Na^+^ currents were filtered at 10 kHz and digitized at 50 kHz. L-type Ca^2+^ currents were filtered at 2 kHz and digitized at 10 kHz. Cell capacitance was determined by integration of the current transient evoked from −80 mV to −70 mV using Clampex 8.0 (Molecular Devices, Foster City, CA). All current-voltage (*I*-*V*) curves were fitted using the following equation:

(1) $$\begin{array}{c} I=G_{\max}*\frac{\left(V-V_{\text{rev}}\right)}{\left(1\;+\;\exp\left[\left(V_{G}-V\right)/k_{G}\right]\right)}, \end{array}$$

where *I* is the current for test potential *V*, *V*_rev_ is the reversal potential, *G*_max⁡_ is the maximum inward (either Na^+^ or Ca^2+^) conductance, *V*_*G*_ is the half-maximal activation potential, and *k*_*G*_ is the slope factor. Conductance-voltage (*G*-*V*) relationships for either Na^+^ or L-type Ca^2+^ currents were derived from the *I*-*V* data using:

(2) $$\begin{array}{c} G\left(V\right)=\frac{I\left(V\right)}{\left(V-V_{\text{rev}}\right)}, \end{array}$$

where the *V*_rev_ value for Na^+^ or Ca^2+^ in each cell was taken from (1). The average conductance values were subsequently fit with the following equation:

(3) $$\begin{array}{c} \;\frac{G}{G_{\max}}=\frac{1}{\left(1+\exp\left[-\left(V-V_{1/2}\text{act}\right)/k\right]\right)}, \end{array}$$

where *G* is the conductance for test potential *V*, *G*_max⁡_ is the maximal cation conductance, *V*_1/2_act is the half-maximal activation potential, and *k* is the slope factor. Steady-state inactivation curves for Na^+^ currents were fit by the following equation:

(4) $$\begin{array}{c} \frac{I}{I_{\max}}=\frac{1}{\left(1+\exp\left[-\left(V-V_{1/2}\text{inact}\right)/k\right]\right)}, \end{array}$$

where *I* is the current amplitude for test potential to 0 mV following a 300 ms prepulse to potential *V*, *I*_max⁡_ is current amplitude evoked by a test depolarization to 0 mV following a 300 ms prepulse to −110 mV, *V*_1/2_act is the half-maximal inactivation potential, and *k* is the slope factor.

### 2.3. Pharmacology

Dantrolene (Sigma no. D9175) was dissolved in dry DMSO to make a 20 mM stock solution, diluted to 10 *μ*M, and sonicated just prior to use. Myotubes were exposed to Dantrolene in the bath solution (~25°) for 10 to 30 minutes. Dantrolene was stored and used in the dark.

### 2.4. Analysis

 Figures were made using the software program SigmaPlot (versions 7.0 and 11.0, SSPS Inc., Chicago, IL). All data are presented as mean ± SEM. Statistical comparisons were by unpaired, two-tailed *t*-test, with *P* < 0.05 considered significant.

## 3. Results

### 3.1. Dantrolene Does Not Affect the Fast Skeletal Muscle Na^+^ Current

**Figure 1:**
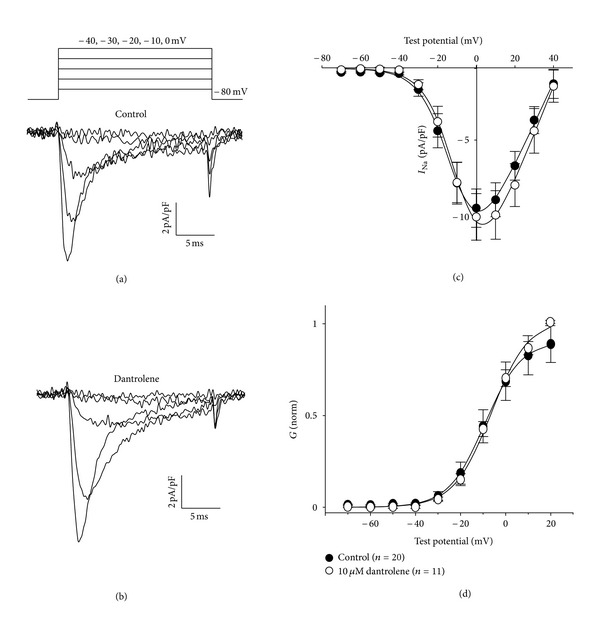
Dantrolene does not affect the *I*-*V* relationship in of the fast skeletal muscle Na^+^ current. Representative recordings of skeletal *I*_Na_ elicited by 20 ms depolarizations from the steady holding potential of −80 mV to the indicated test potentials (illustrated at *top*) are shown for untreated control myotubes (a) and myotubes exposed to 10 *μ*M Dantrolene (b). (c) Comparison of peak *I*-*V* relationships for control (*⚫*; *n* = 20) and Dantrolene-treated (*⚪*; *n* = 11) myotubes. Currents were evoked at 0.1 Hz by test potentials ranging from −70 mV through +40 mV in 10 mV increments. Peak current amplitudes were normalized by linear cell capacitance (pA/pF). Smooth *I*-*V* curves were fit by (1) (see “Section 2”) with the following respective parameters for control and Dantrolene-treated groups: *G*_max⁡_ = 305 ± 43 and 355 ± 44 nS/nF; *V*_1/2_ = −9.8 ± 1.7 and −7.4 ± 1.3 mV; *k* = 6.9 ± 0.3 and 7.2 ± 0.2 mV. (d) comparison of conductance-voltage relationships for control and Dantrolene-treated myotubes. The average normalized conductance values (derived from *I*-*V* data using (2); see Section 2) were fit by (3) with the following respective parameters for control and Dantrolene-treated groups: *V*_1/2_ = −9.9 ± 1.8 and −5.9 ± 3.0 mV; *k* = 6.9 ± 0.3 and 7.9 ± 1.0 mV. Throughout, data are given as mean±SEM, with the numbers in parentheses indicating the number of myotubes tested. For all the data given, the calculated average voltage error was <5 mV.

The whole-cell patch clamp technique [22] was employed to test directly whether Dantrolene affects the fast skeletal muscle Na^+^ current in cultured myotubes (which is conducted by a combination of Na^+^ channel isoforms) [23]. With 5 mM external Na^+^ as the charge carrier, myotubes produced robust, rapidly-activating and -inactivating inward currents (Figure 1(a)). Myotubes exposed to Dantrolene (10 *μ*M) for greater than 10 minutes also produced Na^+^ current with similar amplitude and kinetics (Figure 1(b)). As shown in Figure 1(c), the *I*-*V* relationships obtained in the absence and presence of Dantrolene displayed no significant differences in average peak current density (−9.8 ± 1.3 pA/pF, *n* = 20 versus −10.4 ± 1.6 pA/pF, *n* = 11, resp.; *P* > 0.05). Likewise, fitting of the average conductance values (derived from the *I*-*V* data using the reversal potential for each individual cell; see Section 2) revealed little difference in the voltage-dependence of activation (*V*_1/2_act = −9.9 ± 1.8 mV for untreated versus −5.9 ± 3.0 mV for Dantrolene-treated, resp.; *P* > 0.05 *t*-test; Figure 1(d)).

**Figure 2:**
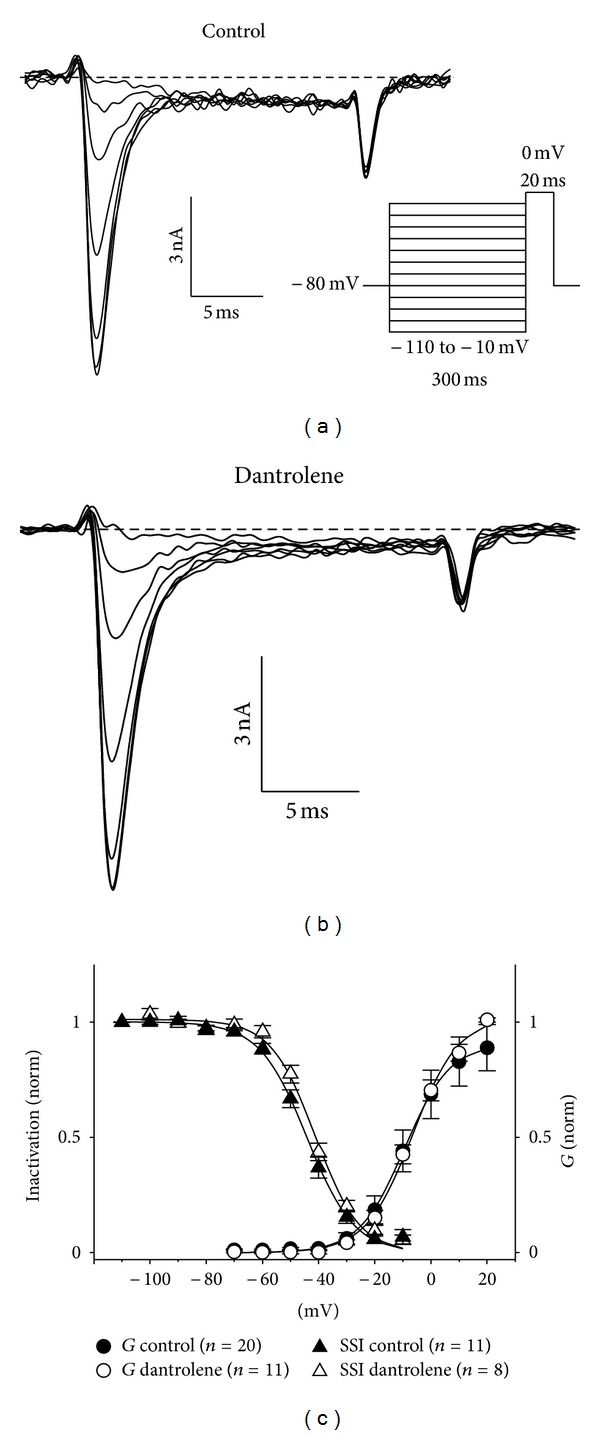
Dantrolene does not affect steady-state inactivation of the fast skeletal muscle Na^+^ current. Representative recordings of skeletal Na^+^ current elicited by 20 ms depolarizations to 0 mV immediately following 300 ms prepulses ranging from −110 mV to −10 mV are shown for untreated control myotubes (a) and myotubes exposed to 10 *μ*M Dantrolene (b). The voltage protocol is not drawn to scale. (c) Voltage-dependence of inactivation for control (*⚫*; *n* = 11) and Dantrolene-treated (*⚪*; *n* = 8) myotubes. Inactivation was fit by (4) (see Section 2) with the following respective parameters for control and Dantrolene treated groups: *V*_1/2_ inact = −44.3 ± 1.6 and −41.2 ± 1.1 mV; *k* = −8.4 ± 0.9 and −7.7 ± 0.3 mV. Inactivation curves are superimposed with activation curves shown in Figure 1 to illustrate window current.

Next, the voltage protocol illustrated at the inset of Figure 2(a) was employed to determine whether Dantrolene influences steady-state inactivation of the fast Na^+^ current. Specifically, 300 ms prepulses (ranging from −110 mV to −10 mV) were applied immediately prior to a 20 ms test depolarization to 0 mV. Steady-state inactivation was normalized to the measured current amplitude evoked by the test depolarization to 0 mV following a prepulse to −110 mV. As shown in Figures 2(a)–2(c), steady-state inactivation of the fast Na^+^ current in the presence of Dantrolene was nearly indistinguishable from that observed in untreated myotubes (*V*_1/2_inact = −41.2 ± 1.1;  *n* = 8 versus −44.6 ± 1.6; *n* = 11, resp.; *P* > 0.05). Superimposition of the steady-state inactivation curves with conductance-voltage relationships revealed minimal window current with no obvious differences in between control and Dantrolene-treated groups (Figure 2(c)).

**Figure 3:**
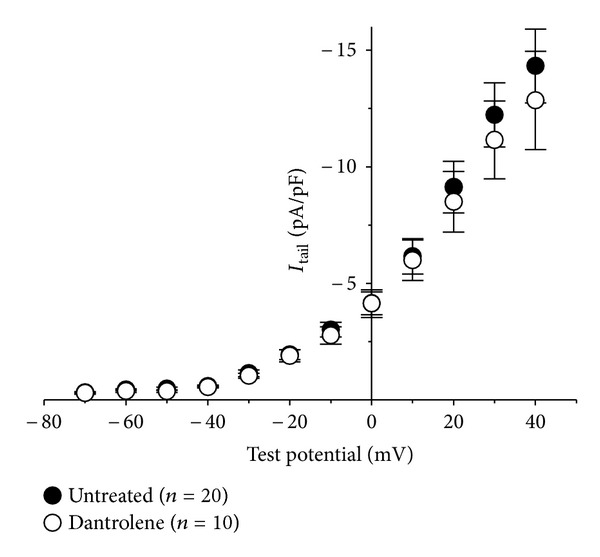
Tail current analysis. (a) Comparison of peak *I*-*V* relationships for control (*⚫*; *n* = 20) and Dantrolene-treated (*⚪*; *n* = 10) myotubes. Tail currents were elicited by repolarization from the indicated test potential to −80 mV. Currents were evoked at 0.1 Hz by test potentials ranging from −70 mV through +40 mV in 10 mV increments. Peak tail current amplitudes were normalized by linear cell capacitance (pA/pF).

In the representative dantrolene-treated cells shown in Figures 1 and 2, there appears to be a reduction in tail current amplitude. In both cases, tail current is reflective of the activation phase of L-type Ca^2+^ current and a lesser contribution of residual Na^+^ current. Analysis of these tail currents indicated that there is a slight, but not quite significant, difference in the peak amplitude of the tail currents at more depolarized test potentials (−12.8 ± −2.1 pA/pF; *n* = 10 versus 14.3 ± 1.6 pA/pF; *n* = 20, resp.; *P* > 0.05; Figure 3). When a contribution from residual Na^+^ current is considered, this difference may be more substantial.

**Figure 4:**
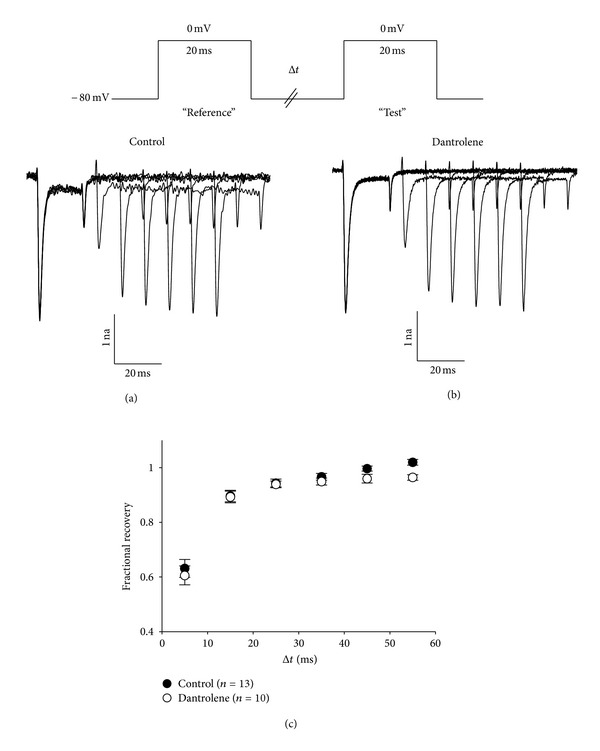
Dantrolene does not affect the recovery of the fast skeletal muscle Na^+^ current from inactivation. Representative recordings of skeletal Na^+^ current elicited by two 20 ms depolarizations from −80 mV to 0 mV separated by time intervals ranging from 5 ms to 55 ms in 10 ms increments are shown for untreated control myotubes (a) and myotubes exposed to 10 *μ*M Dantrolene (b). (c) Summary of results.

The ability of Dantrolene to alter the recovery of the fast Na^+^ current from inactivation was tested using the voltage protocol illustrated at the *top *of Figure 4, in which 20 ms reference pulses evoked by depolarizations from −80 mV to 0 mV were followed by test pulses from −80 mV to 0 mV at intervals increasing in 5 ms increments. Recovery from inactivation was assessed as the fraction of the fast Na^+^ current evoked by the test pulse relative to that evoked by the preceeding reference pulse. In these experiments conducted in wild-type myotubes, the fractional recovery of inactivation was virtually identical for the fast Na^+^ current in the absence (*n* = 13; Figures 4(a) and 4(c)) or in the presence of 10 *μ*M Dantrolene (*n* = 10; Figures 4(b) and 4(c)).

### 3.2. Inhibition of L-Type Currents by Dantrolene Requires Expression of RyR1

**Figure 5:**
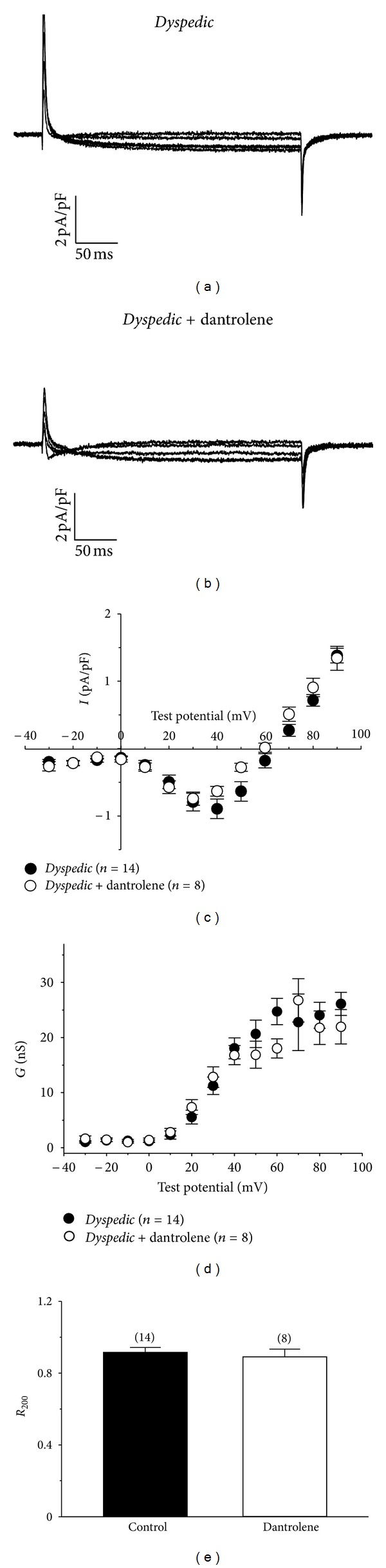
Skeletal L-type Ca^2+^ currents are little affected by Dantrolene in the absence of RyR1. Representative current families evoked from −80 mV to 0, 10, 20, 30, and 40 mV for *dyspedic *myotubes treated with DMSO vehicle (a) or 10 *μ*M Dantrolene (b) for >10 minutes at ~25°C. In the case of the latter, the step from −80 mV to 0 mV evoked some T-type current visible at the beginning of the pulse. Peak *I*-*V* relationships are shown in (c). Smooth *I*-*V* curves were fit by (1) (see “Section 2”) with the following respective parameters for control (*n* = 8) and Dantrolene-treated (*n* = 14) groups: *G*_max⁡_ = 57 ± 6 and 43 ± 5 nS/nF; *V*_1/2_ = 36.6 ± 4.5 and 28.1 ± 2.2 mV; *k* = 10.9 ± 1.6 and 11.8 ± 2.0 mV. No significant (*P* > 0.05, unpaired *t*-test) differences were observed amongst the fit parameters. (d) Comparison of conductance-voltage relationships for control and Dantrolene-treated *dyspedic* myotubes. The average conductance values (derived from *I*-*V* data using (2); see Section 2). (e) Inactivation summary. *R*_200_ = fraction of the peak current remaining at the end of the 200 ms test depolarization to +30 mV.

Dantrolene inhibits voltage dependent Ca^2+^ entry in normal myotubes by shifting the voltage dependence of Ca_V_1.1 gating to more depolarized potentials [19]. Since RyR1 and Ca_V_1.1 exist in a macromolecular complex supported by protein-protein interactions [24], Dantrolene-induced conformational rearrangements of RyR1 might influence conformational changes of Ca_V_1.1 that are involved in channel gating. To test this idea, L-type currents were recorded from *dyspedic *(RyR1 null) myotubes. As previously shown for *dyspedic *myotubes [2–5], L-type currents recorded from these cells were small (−0.74 ± 0.10 pA/pF at +30 mV, *n* = 8) and had more rapid activation kinetics than L-type currents typically observed in normal myotubes (Figure 5(a)) [3–5]. Exposure to Dantrolene had little effect on the amplitude (−0.79 ± 0.13 pA/pF, *n* = 14; *P* > 0.05, unpaired *t*-test) or activation kinetics of *dyspedic* L-type currents (Figure 5(b)). Importantly, no depolarizing shift in the *I*-*V* relationship was observed (*V*_1/2_ act = 36.6 ± 4.5 mV and 28.1 ± 2.2 mV for control and dantrolene-treated, resp., *P* > 0.05; Figure 5(c)). However, the reversal potential for L-type currents in dantrolene-treated myotubes displayed a slight hyperpolarizing shift relative to L-type currents in control myotubes (*V*_rev_ = 59.5 ± 1.7 mV and 64.0 ± 2.0 mV, resp., *P* > 0.05, unpaired *t*-test; Figure 5(c)). For this reason, the conductance-voltage relationships for control and Dantrolene-treated *dyspedic *myotubes conductance relationships were derived using (2) and were found to be similar (Figure 5(d)).

In an earlier work, Szentesi and colleagues [25] reported that Dantrolene slows inactivation of L-type current in adult rodent fibres. To investigate whether such an effect of Dantrolene on Ca_V_1.1 inactivation requires RyR1, inactivation of the L-type current was quantified in control and Dantrolene-treated *dyspedic* myotubes as the *R*_200_ value (fraction of the peak current remaining at the end of the 200 ms test depolarization to +30 mV). As summarized in Figure 5(e), the *R*_200_ values for control (0.91 ± 0.03,  *n* = 14) and Dantrolene-treated (0.89 ± 0.04, *n* = 8) *dyspedic* myotubes were not significantly different (*P* > 0.05, unpaired *t*-test). Taken together, the inability of Dantrolene to modulate the skeletal L-type current in the absence of RyR1 supports the idea that these previously observed effects of Dantrolene on the L-type current [19, 25] in normal muscle resulted from altered conformational coupling between RyR1 and Ca_V_1.1. 

## 4. Discussion

It is widely accepted that Dantrolene inhibits excitation-contraction (EC) coupling without greatly affecting the ability of skeletal muscle fibers to conduct action potentials [26]. However, recent evidence demonstrating that Dantrolene inhibits skeletal muscle L-type Ca^2+^ current and/or charge movement attributed to Ca_V_1.1 [19, 25, 27] has raised the possibility that Dantrolene may produce inhibition of Ca_V_1.1 by altering the membrane environment. In order to probe depression of plasma membrane excitability as the mechanism of L-type channel inhibition, the effects of Dantrolene on various facets of the skeletal muscle Na^+^ current were investigated. Thus, exposure to Dantrolene produced no significant effect on the average peak current density, voltage dependence of activation, voltage dependence of inactivation, or recovery from inactivation of the Na^+^ current in developing myotubes (Figures 1–3). The inability of Dantrolene to modulate these parameters of the Na^+^ current in this preparation is consistent with the idea that the Dantrolene-induced inhibition of Ca_V_1.1 observed in previous studies [19, 25, 27] most likely was a consequence of Dantrolene interacting with the EC coupling apparatus at plasma membrane-SR junctions, rather than a general nonspecific depression of plasma membrane excitability.

The observation that Dantrolene did not greatly alter the amplitude, voltage dependence, or inactivation kinetics of L-type Ca^2+^ currents in *dyspedic* myotubes (Figure 5) lends support to the idea that the inhibition of L-type current by dantrolene is unlikely a consequence of a direct interaction of Dantrolene with Ca_V_1.1 channels. One caveat to this interpretation is that dantrolene may selectively interact with high P_0_ states of Ca_V_1.1 that only occur with the influence of RyR1 [2, 3]. In either case, the data presented in Figure 5 indicate that expression of RyR1 is necessary for Dantrolene's inhibitory effects on Ca_V_1.1 and that Dantrolene alters conformational coupling between the two channels.

**Figure 6:**
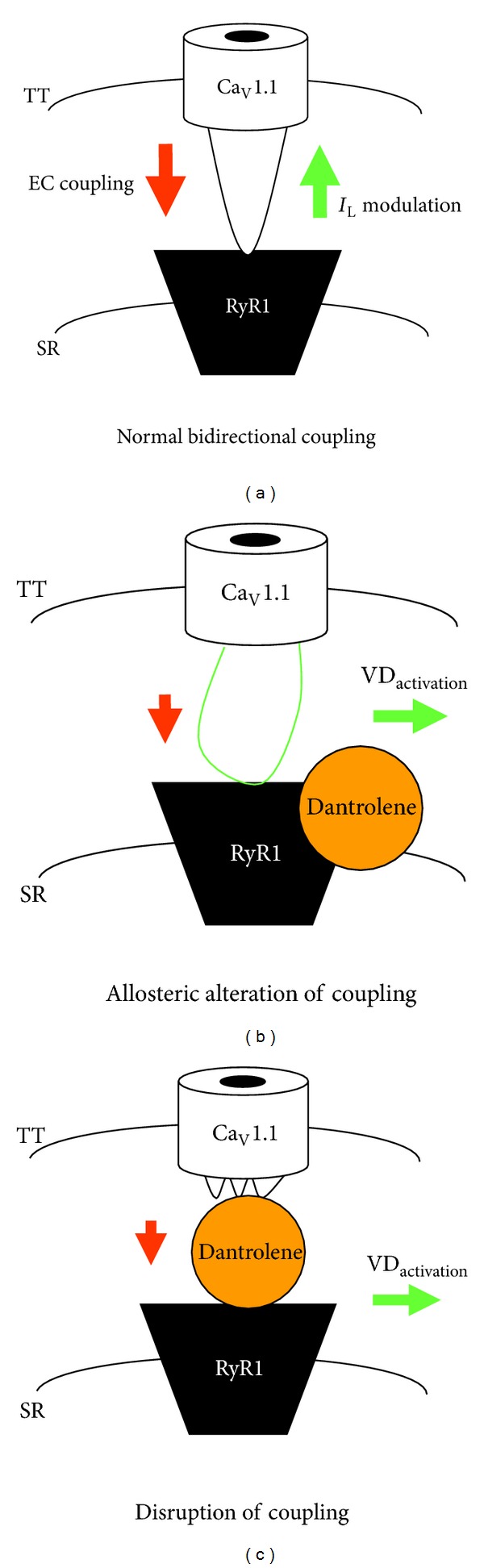
Potential models for Dantrolene-mediated inhibition of L-type Ca^2+^ current and excitation-contraction coupling in skeletal muscle. (a) A simplified model illustrating bidirectional coupling between RyR1 and Ca_V_1.1, the skeletal muscle L-type Ca^2+^ channel [2]. The *red *arrow represents orthograde signal transmitted from Ca_V_1.1 to RyR1 that engages SR Ca^2+^ release (i.e., EC coupling). The *green* arrow represents the retrograde communication transmitted from RyR1 to Ca_V_1.1 that enhances channel P_0_ and modifies L-type current activation kinetics. Panels B and C show two possible mechanisms by which Dantrolene alters Ca_V_1.1 function. (b) Binding of Dantrolene to RyR1 causes conformational changes in RyR1 that reduce SR Ca^2+^ release (represented by small *red *arrow) and shifts the activation of L-type currents to more depolarizing test potentials by altering retrograde contacts between RyR1 and Ca_V_1.1 (represented by horizontal *green *arrow). (c) Alternatively, dantrolene-induced inhibition of SR Ca^2+^ release (again represented by small *red *arrow) and alterations in L-type current activation (again represented by horizontal *green *arrow) may be a consequence of disruption of interactions between RyR1 and Ca_V_1.1 that are critical for bidirectional communication between the two channels.

Figure 6(a) shows a simplified model of “normal” bidirectional coupling between Ca_V_1.1 and RyR1 after [2] where the *green* arrow and the *red* arrow represent orthograde (i.e., skeletal-type EC coupling) and retrograde (i.e., modulation of L-type current by RyR1) signaling, respectively. The precise mechanism of Dantrolene-induced inhibition of orthograde coupling is area of controversy. A body of evidence points to a mechanism in which Dantrolene interferes with orthograde signaling by inhibiting Ca^2+^ release from the SR. This view is based largely on the observations that radiolabelled Dantrolene binds to RyR1 directly [28, 29] and inhibits RyR1-mediated Ca^2+^ efflux from SR vesicles [30, 31]. However, other investigators have found little effect of Dantrolene on the single channel properties of RyR1 in reconstituted lipid bilayers [18, 25]. 

Although my present observations do not resolve the issue of how Dantrolene inhibits orthograde coupling, they do provide support for the idea that Dantrolene's effect on retrograde signaling stems from a junctional interaction of Dantrolene with the intact (i.e., RyR1-containing) EC coupling macromolecular complex. These findings are incorporated into the two hypothetical models shown in Figures 6(b) and 6(c). In the first model (Figure 6(b)), Dantrolene would alter Ca_V_1.1 gating indirectly by inducing allosteric rearrangements in RyR1. This model is based on the notion that agents that alter the activity of orthograde coupling by altering the functional state of RyR1 may affect retrograde signaling and vice-versa. For example, application of a high concentration of ryanodine (≥200 *μ*M) not only attenuates EC coupling by locking RyR1 in a nonconducting state [32, 33], but also causes hyperpolarizing shifts in skeletal muscle L-type current activation [34, 35] and in charge movement [35]. Similarly, Dantrolene-induced allosteric changes in the myoplasmic region of RyR1 may affect Ca_V_1.1 in such a way that causes a depolarizing shift in L-type current activation. 

Another potential mechanism for the inhibition of Ca_V_1.1 gating is that Dantrolene could disrupt the protein-protein interactions between RyR1 and the Ca_V_1.1 heteromultimer that support bidirectional signaling (illustrated in Figure 6(c)). Within the context of this model, Dantrolene not only would impair EC coupling by blocking the transient interaction between the Ca_V_1.1 channel and RyR1 but also would cause inhibition of the L-type current by removing the influence of RyR1 on Ca_V_1.1 gating.

In summary, the anti-MH drug Dantrolene has little effect on the biophysical properties of the Na^+^ current or L-type Ca^2+^ current in developing skeletal muscle harvested from normal mice or mice lacking RyR1, respectively. These results indicate (1) that a general depression of plasma membrane excitability seems not to be responsible for inhibition of skeletal L-type Ca^2+^ current by Dantrolene, and (2) that RyR1 expression is necessary for the effect(s) of Dantrolene on the L-type current. Thus, this study reveals useful information towards the mechanism of Dantrolene's effect on Ca^2+^ currents mediated by Ca_V_1.1.
